# Supplementary material for: Confident Body, Confident Child: Outcomes for Children of Parents Receiving a Universal Parenting Program to Promote Healthful Eating Patterns and Positive Body Image in Their Pre-Schoolers—An Exploratory RCT Extension
Source: Int J Environ Res Public Health. 2020 Jan 31;17(3):891. doi: 10.3390/ijerph17030891 (PMC7037269; doi:10.3390/ijerph17030891)
Supplement: Supplementary file 1 [file ijerph-17-00891-s001.pdf]

Supplementary File 1

In the current study, only participants who completed all measurement occasions up to and including 12-months follow-up, were invited to participate. The eligible sample included 252 parents (n=58 CBCC resource + workshop, n=77 CBCC resource only, n=61 nutrition control, n=56 waitlist control) who were reporting on 157 girls aged 4-6 years (62%) and 95 boys (38% boys) aged 4-6 years. Of those eligible to participate in the current study, 115 (46%) parents consented to their child being interviewed, although only 89 children completed the measures with sufficient data to be included in analyses.

Participants were 58 girls and 31 boys of parents from all four groups of the established RCT cohort (CBCC resource + workshop n=27, CBCC resource only n=26, nutrition control n=18 and waitlist control n=18).

To ascertain whether the sample used in the current study was representative of the sample in the original RCT, two groups of parents were compared on a range of parent and child characteristics at baseline. Across the 252 eligible participants, those who went on to complete the 18-month follow-up occasion child interview, were compared with those who were eligible to participate but did not complete the 18-month assessment (see Table 1). Across all variables, these two groups were not statistically significantly different from each other, suggesting that the sample used in the current study is likely representative of the original RCT cohort.

Table S1. Characteristics of Parent Participants at baseline by Intervention Group according to 18-month completers versus eligible non-completers.

	CBCC Workshop + Resource[a]	CBCC Resource[b]	Nutrition Resource[c]	Waitlist control[d]	Total sample	p-value[e]
Participant sample (n)						0.069*
At baseline (n)	77	106	86	76	345	
Eligible for 18m study (completed 12m)	58	77	61	56	252	
Eligible non-completers at 18m	31	51	43	38	163	
18m completers (n with child interview)	27	26	18	18	89	
Age at baseline						0.655
Eligible non-completers (M, SD)	38.1 (2.9)	37.6 (4.4)	37.9 (4.4)	37.8 (3.5)	37.76 (3.9)	
18m completers (M, SD)	36.8 (3.9)	38.0 (3.7)	38.2 (3.4)	37. 0 (4.6)	37.34 (4.0)	
Gender						0.235
Eligible non-completers (% female)	93.5	100	95.3	94.7	96.3	
18m completers (% female)	100	100	94.4	100	98.9	
Ethnicity						0.815
Eligible non-completers (% other than Australian)	48.4	33.3	44.2	47.4	42.0	
18m completers (% other than Australian)	33.3	30.8	38.9	44.4	36.0	
SEIFA						0.731
Eligible non-completers in lowest 20% (% yes)	0.0	5.9	9.3	2.6	4.3	
18m completers in lowest 20% (% yes)	0.0	3.8	5.6	0.0	2.2	
Eligible non-completers in highest 10% (% yes)	22.6	19.6	16.3	18.4	19.0	
18m completers in highest 10% (% yes)	15.6	22.2	14.3	31.8	20.2	
Relationship Status						0.398
Eligible non-completers Married/Defacto/Partnered (% yes)	100	96.1	95.3	97.3	96.6	

18m completers Married/Defacto/Partnered (% yes)	96.3	100	100	100	99.0	
Highest Education						0.263
Undergraduate University Degree (% yes) Eligible	51.6	47.1	44.2	42.9	42.9	
Undergraduate University Degree (% yes) 18m completers	50.0	40.7	28.5	54.5	42.7	
Postgraduate University Degree (% yes) Eligible	29	35.3	32.6	60.5	39.5	
Postgraduate University Degree (% yes) 18m	34.4	44.4	66.6	36.4	44.9	
Occupational Status						0.698
Eligible non-completers Home duties only (% yes)	32.3	25.5	25.6	15.8	24.5	
18m completers Home duties only (% yes)	29.6	30.8	38.9	16.7	26.4	
Eligible non-completers Work/study only (% yes)	54.8	56.9	55.8	52.6	55.2	
18m completers Work/study only (% yes)	48.1	50.0	44.4	61.1	51.0	
Eligible non-completers Work+Home (% yes)	12.9	17.6	18.6	31.6	18.6	
18m completers Work+Home (% yes)	22.2	19.2	16.7	22.2	22.5	
Weight Status[f]						0.306
Eligible non-completers % BMI<18.5	3.2	4.0	0	2.6	2.4	
18m Completers % BMI<18.5	0	3.1	0	9.0	2.2	
Eligible non-completers % BMI 18.5-24.9	58.1	62.7	53.5	57.7	58.0	
18m Completers % BMI 18.5-24.9	53.12	74.0	61.9	45.5	57.3	
Eligible non-completers % BMI 25-30	32.3	27.5	34.9	36.8	32.1	
18m Completers % BMI 25-30	25	14.8	33.3	31.8	27.0	
Eligible non-completers % BMI>30	6.5	5.9	11.6	5.3	7.4	
18m Completers % BMI>30	21.9	7.4	4.7	13.6	13.4	
EDE-Q Total Score[g]						0.643
Eligible non-completers (M,SD)	2.4 (1.5)	1.9 (1.3)	2.4 (1.5)	2.1 (1.4)	2.19 (1.4)	
18m completers (M,SD)	2.9 (1.62)	2.3 (1.5)	1.8 (1.4)	2.9 (1.1)	2.48 (1.5)	
BAQ[h]						0.893
Eligible non-completers (M,SD)	39.3 (11.5)	35.7 (10.6)	40.4 (12.5)	37.8 (11.7)	38.0 (11.6)	
18m completers (M,SD)	44.2 (11.2)	38.3 (12.1)	36.18 (11.7)	43.4 (10.9)	40.5 (11.7)	

SATAQ Internalization[i]						0.167
Eligible non-completers (M,SD)	21.1 (9.2)	20.4 (7.9)	19.5 (9.5)	20.1 (7.5)	20.22 (8.4)	
18m completers (M,SD)	19.4 (6.8)	20.6 (7.2)	17.3 (8.4)	20.2 (7.0)	19.72 (7.4)	
Child Age in Years at Baseline						0.852
Eligible non-completers (M,SD)	4.2 (.95)	4.1 (1.1)	3.8 (1.2)	3.9 (.92)	4.03 (1.0)	
18m completers (M,SD)	3.7 (.97)	4.3 (.94)	4.3 (0.93)	3.9 (1.13)	4.03 (1.0)	
Child Gender						0.465
Eligible non-completers % female	61.3	62.7	53.5	65.8	60.49	
18m Completers % female	59.3	69.2	61.1	72.2	65.2	
Child BMIz[j]						0.758
Eligible non-completers (M, SD)[j]	0.45 (1.1)	0.52 (1.2)	0.25 (1.2)	0.36 (1.1)	0.40 (1.1)	
18m Completers (M, SD)	0.82 (1.1)	0.23 (1.2)	0.97 (1.0)	0.61 (0.79)	0.63 (1.1)	

Note: P-values based on chi-square tests for categorical variables and analysis of variance for continuous variables. SEIFA: Socio-Economic Indexes for Areas. EDE-Q: Eating Disorder Examination Questionniare, BMIz WHO Cat: World Health Organization Body Mass Index z-score Categorization.

a = All parents attended one two-hour face-to-face workshop and received the Confident Body, Confident Child Resource Pack (Parent Book, Extended Family Book, *Shapesville* children's storybook, Do/Don'ts poster, access to the confidentbody.net website).

b = All parents received the Confident Body, Confident Child Resource Pack via post.

c = All parents received the Happy Healthy Kids for Life nutrition booklet via post.

d = All parents had delayed receipt of both the Confident Body, Confident Child Resource Pack and the Happy Healthy Kids for Life nutrition booklet via post, until after all four evaluation questionnaires were completed.

e = Statistical significance testing was based on the total sample of parents who were eligible to participate at 18-months (n=252; Eligible sample) comparing those who did complete the follow-up time point with a child interview (n=89; 18-month completers) versus those who did not complete (n=163, non-completers). Although raw data are provided broken down by intervention group, due to small cell sizes statistical analyses were restricted to the 'total sample' values only (i.e., completed versus did not complete) and not based on values broken down by intervention group.

f = height and weight were self-reported by parents and used to calculate Body Mass Index. Data was missing for 7 participants.

g = Excluding all women who indicated they were currently pregnant or had given birth in the last 6 months.

h = Body Attitudes Questionnaire, a measure of body dissatisfaction

i= Sociocultural Attitudes Towards Appearance Questionnaire, Internalization subscale

j= *Height*/weight data was missing for 20 children. Parents were asked to report whether measures were an estimate or exact measure, with 68% reporting an exact measure.

* Chi-square test for independence: χ^2 (3, n=252) = -.102; comparing frequency across groups (A, B, C, D) of those who completed 18-month follow-up versus those who were eligible but who did not complete.
